# Editorial: Bacterial persister cells in the food industry

**DOI:** 10.3389/fmicb.2025.1673218

**Published:** 2025-08-18

**Authors:** Pedro Rodríguez-López, Filippo Giarratana, Luca Nalbone

**Affiliations:** ^1^Department of Animal and Food Science, Area of Nutrition and Bromatology, Faculty of Veterinary Sciences, Universitat Autònoma de Barcelona, Bellaterra (Cerdanyola del Vallès), Spain; ^2^Department of Veterinary Sciences, University of Messina, Polo Universitario dell'Annunziata, Messina, Italy

**Keywords:** antimicrobial resistance, bacterial persistence, biofilms, food quality and safety, food industry, foodborne pathogens, specific spoilage organisms

In recent years, there has been a general increase in awareness regarding the prevalence of *Bacterial persister cells in the food industry*. These are defined as a subset of cells that, upon exposure to external stressors such as antimicrobials or radiation, enter a non- or slow-growing state. This enables them to withstand and survive harsh environmental conditions, posing a significant challenge to effective control measures (Serrano et al., 2025). Given the potential risks to food safety associated with this phenotype, it is of the upmost importance to understand the key determinants and underlying molecular mechanisms triggering bacterial cells to enter the persister state, as well as their survival strategies.

**Figure 1:**
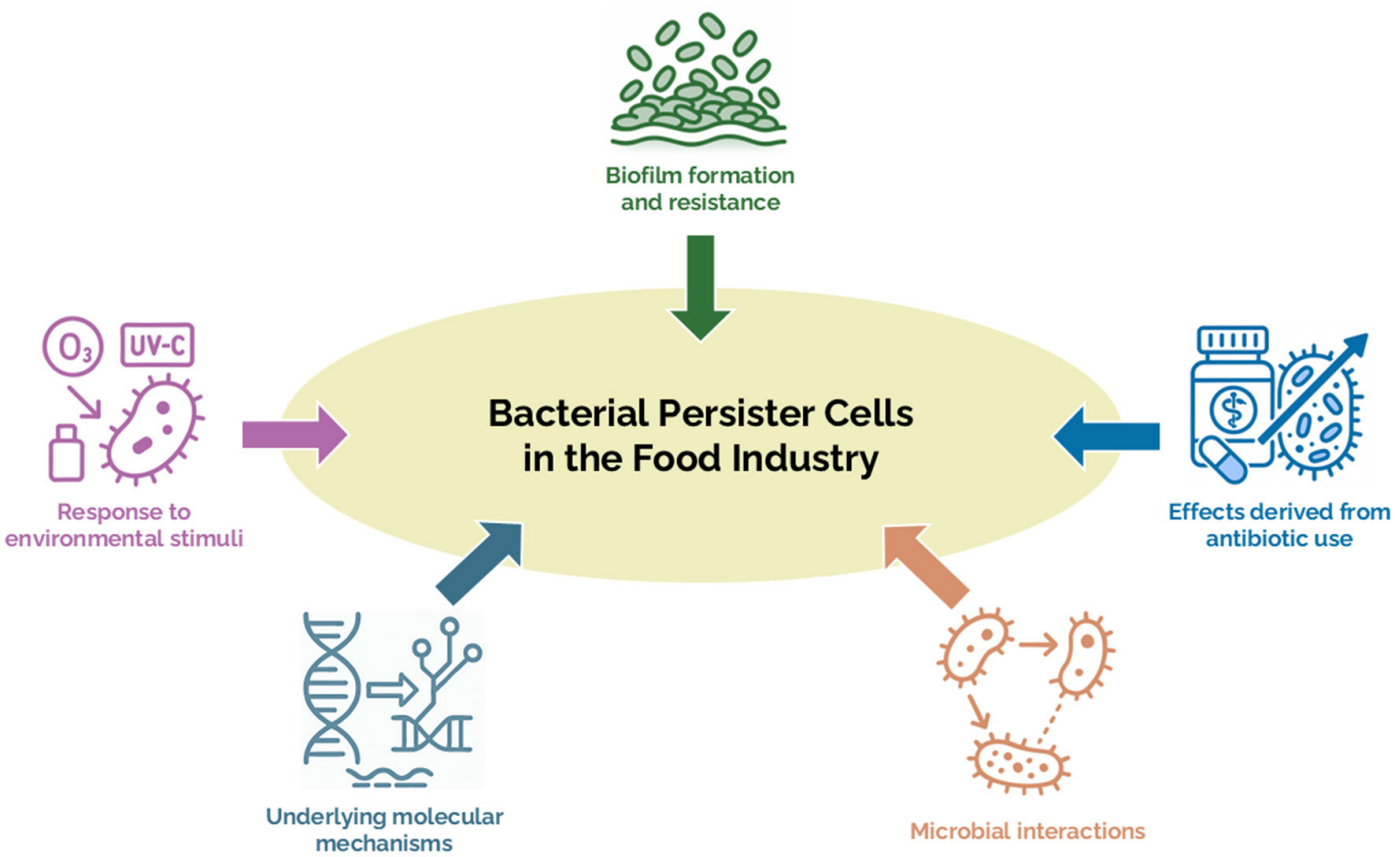
Main thematic areas addressed by the articles published in the Research Topic.

Hence, a global call for contributions was launched for a Research Topic entitled “*Bacterial persister cells in the food industry*.” As guest editors, we hereby present a Research Topic of seven original research articles addressing different aspects of persisters, as illustrated in Figure 1.

Kim et al. evaluated the effects of streptomycin on *Erwinia amylovora*, the aetiological agent of fire blight. The authors demonstrated that exposure to 500 μg·mL^−1^ followed by incubation in distilled water, induced a persister state maintained for up to 48 h before transitioning into a viable-but-not-culturable (VBNC) phenotype, with cell viability sustained for up to 15 days. Resuscitation of both cell variants was achieved after a secondary treatment of either 10 mM glucose, fructose or sorbitol, whereas sucrose and tri-basic copper sulfate did not. Furthermore, they observed that 10 mM oxytetracycline eliminated both subpopulations. These findings underscore *E. amylovora* persistence under antibiotic stress and highlight the need of integrating dormancy-targeted approaches into phytopathogen management schemes.

Similarly, Xiang et al. provided novel insights into the underlying molecular mechanisms by which *Weissella cibaria*, widely used in food fermentation, survive following tetracycline exposure. They found that tetracycline activated the co-transcription of Bro-Xre toxin-antitoxin (TA) bicistronic operon, making free Bro toxin to intracellularly increase, thus down-regulating various metabolic pathways such as the phosphotransferase system, the hexose monophosphate and the Embden-Meyerhof-Parnas pathways, the

tricarboxylic acid cycle, and the oxidative phosphorylation. This produced a metabolic shift leading *W. cibaria* cells to enter into a dormant, tetracycline-resistant state. These results advance our understanding of the role of TA modules as mediators of persisters cells and of the potential risks posed by antibiotic residues in fermentation processes.

In addition to antibiotics, environmental factors can induce foodborne pathogens cells to become persistent (Nalbone et al., 2024). In this context, Chen et al. observed that *Salmonella enterica* present on raw almonds (RWAs) and fresh-cut leafy greens (FCLGs) better withstood 500 μW·cm^−2^ UV-C treatments for 60 min after being exposed to sub-lethal desiccation, heat-shock, oxidation or acidic conditions, with significant higher *S. enterica* counts observed in RWAs compared to FCLGs. Moreover, the general stress response regulator *rpo*S was shown to be essential for this cross-protection. Finally, non-linear regression modeling and multivariate analysis suggested phenotypic heterogeneity among *S. enterica* persisters, related to different physiological stress adaptations. This work highlights the need to included stress-adapted cells in challenge tests to improve predictive models and risk assessments.

The study of Catania et al. explored the potential of ozone treatments as a sustainable antimicrobial approach against a Research Topic of *Bacillus cereus* and *Bacillus subtilis* isolates previously obtained from an Italian dairy plant. The authors evaluated the effect of 50 ppm ozone exposure on biofilms formed on either polystyrene or stainless steel, as well as on their planktonic counterparts. The outcomes showed that ozone inactivated free-living cells of both species within 6 h, whereas biofilm inactivation was variable, being more effective on *B. subtilis* than *B. cereus*, the latter even showing a biomass increase after treatment, in some strains. Scanning electron microscopy confirmed these findings, revealing species-specific biofilm structural differences that may partly explain the varying ozone effectiveness. This work points out the complexity of biofilm resilience in food-contact surfaces and supports the development of eco-friendly sanitization procedures.

Accompanying microbiota may significantly influence the survival of foodborne pathogens to chlorine-based and quaternary ammonium compounds (QACs) sanitization in polymicrobial biofilms (Rodríguez-López et al., 2022). In this line, Visvalingam et al. demonstrated that environmental bacteria from meat processing plants influenced biofilm formation of a non-biofilm-forming *Escherichia coli* O157:H7 strain, namely EC1934, in dual culture. No significant differences in EC1934 biofilm counts were observed in most dual cultures, except when co-cultured with a particular *E. coli* strain, EC136, that significantly reduced EC1934 counts. Similarly, in EC1934-*Acinetobacter haemolyticus* biofilms higher counts of the latter were obtained. Finally, cleaning and disinfection assays showed greater reduction in mono-species biofilms following QAC-based and chlorine-based sequential treatments than with single-biocide treatments. These findings emphasize the role of accompanying microbiota in biofilm formation and tolerance to food-grade biocides, shielding pathogens from sanitization and promoting their persistence in food-related environments.

The integration of whole genome sequencing (WGS) into outbreak investigations has greatly enhanced the continuous monitoring of pathogenic strains (WHO, 2023). Hence, Carter et al. conducted a comparative study of genomic and phenotypic characteristics associated with the persistence of different *E. coli* O157:H7 strains, including PNUSAE013245 (clade 8) and PNUSAE043864 (clade 2), both associated with romaine lettuce outbreaks in the United States. WGS results revealed strain-specific genomic islands encoding different antimicrobial and heavy metal resistance genes. Biofilm formation was higher among clade 2 strains compared to clade 8 strains. In addition, significant higher survival rates of persisters and VBNC cells in clade 2 strains inoculated in river water were after 14 weeks at 15°C. These observations call attention to consider clade-specific traits when designing persister control interventions in leafy greens production systems.

Using an RNA-Seq approach, Delaporte et al. characterized the transcriptomes of two *Campylobacter jejuni* strains, one aerotolerant and one aerosensitive, under aerobic stress. Authors demonstrated that after exposure to oxygen, the aerotolerant strain exhibited faster and broader gene regulation within 6 h, peaking at 12 h post-exposure, whereas the aerosensitive strain displayed a delayed response peaking at 24 h post-exposure. Both strains upregulated heat shock response genes, e.g., *clp*B, *dna*K, *gro*L, and *gro*S, among others, suggesting their involvement in oxidative stress responses. Additionally, genes related to iron uptake, biofilm formation and ribosomal structure were also differentially expressed. These findings revealed complex mechanisms underlying oxygen resistance of *C. jejuni* and contributing to understanding its persistence in food systems' environments.

Summarizing, this Research Topic presents a comprehensive collection of research articles that highlight key findings on the stimuli, characteristics and molecular mechanisms driving food industry-associated bacteria to adopt the persister phenotype, the subsequent implications for antimicrobial resistance, and their interactions within mono- and multi-species communities. Collectively, the published manuscripts offer valuable insights for researchers in the field, supporting the development of innovative, *ad hoc* strategies to mitigate the risks and impacts associated with bacterial persister cells in food production environments.
